# Comparing Minimally‐Invasive and Open Approaches to Siewert II/III Gastro‐Esophageal Junction Adenocarcinoma—A Systematic Review and Network Meta‐Analysis

**DOI:** 10.1002/wjs.70528

**Published:** 2026-09-06

**Authors:** Connor J. Colson, Matthew G. Davey, Noel E. Donlon

**Affiliations:** ^1^ Royal College of Surgeons in Ireland Dublin Ireland; ^2^ Department of Surgery Our Lady of Lourdes Hospital Drogheda Ireland; ^3^ Department of Surgery St James's Hospital Dublin Ireland

**Keywords:** adenocarcinoma of the gastro‐esophageal junction, laparoscopic surgery, robotic surgery, siewert II, siewert III

## Abstract

**Background:**

The prevalence of adenocarcinoma of the esophagogastric junction has increased, likely driven by lifestyle factors such as obesity, gastro‐esophageal reflux disease, and smoking. However, the long‐term outcomes of different curative surgical approaches have yet to be comprehensively evaluated.

**Objectives:**

To perform a systematic review and meta‐analysis evaluating the surgical approaches to resection of Siewert II/III Adenocarcinoma of the Esophagogastric junction.

**Setting:**

Integration of data from upper gastrointestinal surgical units across the world.

**Methods:**

A systematic review was performed as per PRISMA guidelines. Statistical analysis was performed with MetaInsight.

**Results:**

A total of 16 studies, comprising 4256 patients, were included in the meta‐analysis. The findings suggest that robotic surgical approaches demonstrate equipoise to both laparoscopic and open techniques for resection, with comparable overall survival (OS) at both 3 and 5 years (3‐year OS: OR 1.17, 95% CI [0.10–14.06]; 5‐year OS: OR 0.66, 95% CI [0.39–1.12]). Additionally, a nonsignificant trend toward improved disease‐free survival (DFS) was observed for robotic surgery at both 3 and 5 years (OR 1.17, 95% CI [0.10–14.06] for both time points).

**Conclusion:**

Laparoscopic approaches to surgical resection have proven to be as effective as open surgery for achieving R0 resection. Based on our meta‐analysis results, we can conclude that robotic approaches to resection is equivalent to laparoscopic approaches.

**Prospective Register:**

International Prospective Register of Systematic Reviews (PROSPERO): CRD42024590940

## Graphical abstract

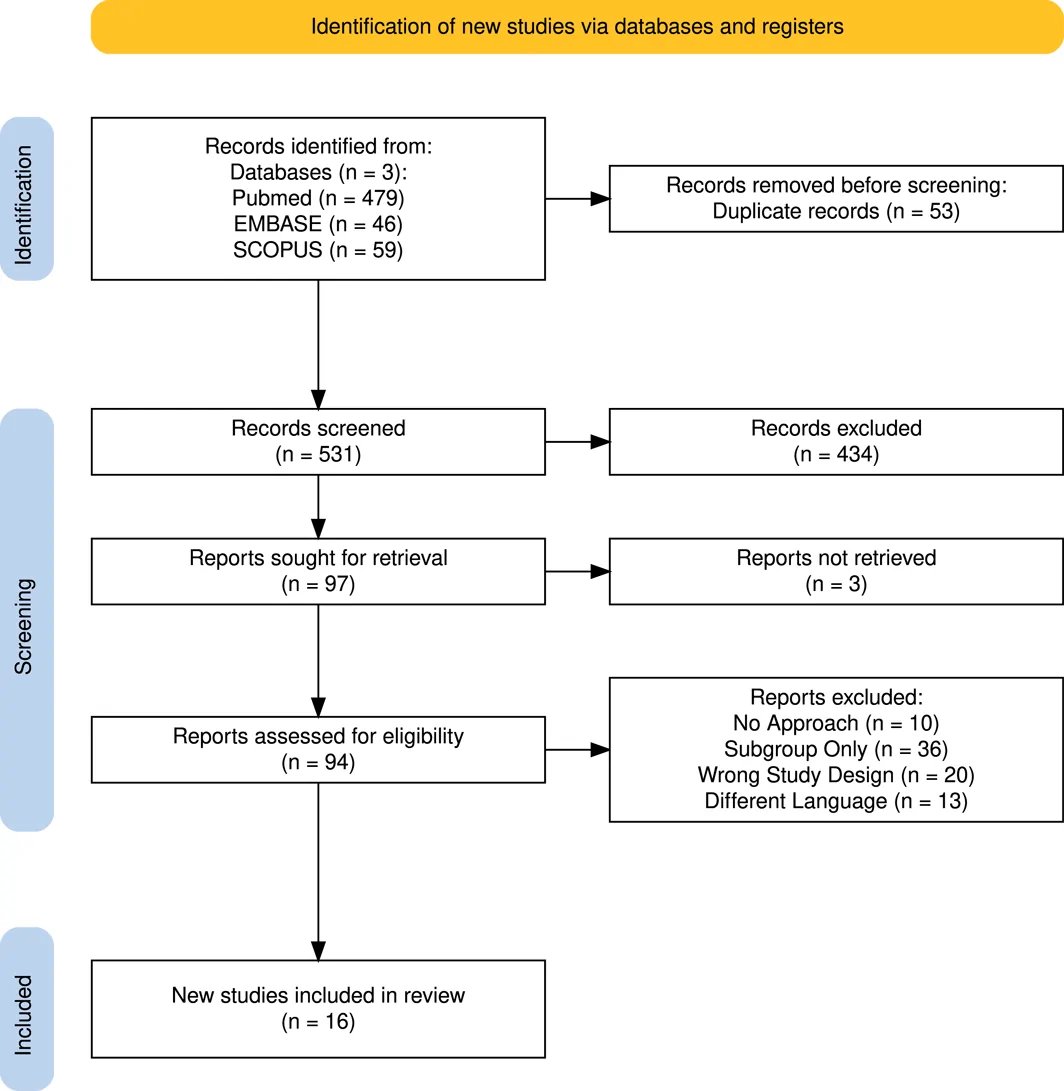

## 1 Introduction

Adenocarcinoma of the gastroesophageal junction (GOJ) is becoming increasingly prevalent in the Western world, a trend which is likely driven by lifestyle‐related risk factors such as obesity, gastroesophageal reflux disease, and smoking [1, 2, 3]. Contemporary management of adenocarcinoma of the GOJ is commonly guided by the Siewert classification system, which delineates specific surgical approaches for each tumor subtype (I–III) [4]. Surgical resection, in tandem with neoadjuvant therapeutics, remains the cornerstone of curative treatment in early to mid‐stage disease [2, 3].

In this review, we focus specifically on gastroesophageal junction (GOJ) tumors, which pose unique diagnostic and therapeutic challenges. Two major classification systems are used to define GOJ cancers: the internationally recognized Siewert classification and the Nishi classification, which is predominantly used in Japan. Both systems aim to guide surgical management; however, there is no universal consensus on the precise anatomical boundaries of these tumors. Overlapping definitions highlight the complex “transitional zone” in which these cancers arise, exhibiting both esophageal and gastric lymphatic drainage patterns—thus complicating surgical planning and decision‐making [5]. This NMA concentrates on Siewert type II and III adenocarcinomas of the esophagogastric junction (AEG). Siewert type II AEG is defined as a tumor located between 1 cm above and 2 cm below the GOJ, often referred to as “true carcinoma of the cardia.” Siewert type III AEG refers to tumors located between 2 and 5 cm below the GOJ, and these are typically classified as gastric cancers [5, 6, 7]. Type II AEG is considered a true junctional tumor, sharing biological and clinical features of both esophageal and gastric adenocarcinomas. Unlike type I and III tumors, there is no universally accepted surgical strategy for type II disease, making its management a subject of ongoing debate [8]. However, these guidelines do not specify the preferred surgical approach in terms of access—that is, open, laparoscopic, or robotic techniques.

The choice of surgical technique—whether open, laparoscopic, or robotic—depends on several factors, including tumor burden, location, the extent of lymphatic involvement, and the preferred method of reconstruction. Although open surgery was historically regarded as the standard of care, the adoption of minimally invasive (laparoscopic or robotic) or hybrid approaches to AEG has become increasingly common. Robotic surgery has gained significant momentum due to its distinct advantages, including enhanced three‐dimensional visualization, improved ergonomic working conditions for the primary operator, and greater precision in instrument manipulation [3].

Previous studies have compared individual surgical modalities for adenocarcinoma of the GOJ, but to date, no comprehensive network meta‐analysis (NMA) has evaluated the safety and efficacy of all three approaches (i.e., robotic, laparoscopic, or open) simultaneously [9, 10]. A network meta‐analysis offers a valuable solution, enabling both direct and indirect comparisons across open, laparoscopic, and robotic techniques to better inform clinical decision‐making. Accordingly, the aim of the current study was to perform a systematic review and NMA of prospective randomized clinical trials (RCTs) to evaluate the surgical, oncological, and survival outcomes of robotic, laparoscopic, and open surgery in the management of Siewert II/III AEG.

## 2 Methods

This study adhered to the Preferred Reporting Items for Systematic Reviews and Meta‐Analyses (PRISMA) guidelines, which were used as a standardized reporting checklist [11, 12]. The review protocol was registered with the Prospective Register of Systematic Reviews (PROSPERO) under the registration number CRD42024590940.

### 2.1 Search Strategy

A comprehensive systematic search was conducted in September 2024 across three major electronic databases: PubMed, EMBASE, and SCOPUS, using a combination of the following search terms: *Siewert*, *esophagogastric junction*, *adenocarcinoma*, and *surgery*. In addition, the reference lists of all included studies were manually screened to identify any additional relevant publications. The final literature search was performed in May 2025.

### 2.2 Selection Criteria

All published RCTs and comparative studies with full‐text manuscripts comparing the outcomes of two or more surgical approaches—robotic, laparoscopic, or open—for Siewert type II/III esophagogastric junction (OGJ) adenocarcinomas were included in this review.

The inclusion criteria were: (1) studies assessing the impact of different surgical approaches (i.e., any two or more of robotic, laparoscopic, or open) on survival outcomes in patients with Siewert II/III OGJ adenocarcinomas, and (2) availability of full‐text manuscripts. Studies were excluded if they met any of the following criteria: (1) studies that did not evaluate the impact of different surgical approaches (robotic vs. laparoscopic vs. open) on either short‐term or long‐term outcomes in patients with Siewert II/III OGJ adenocarcinomas; (2) studies reporting outcomes solely for subgroups; (3) conference abstracts; (4) review articles; (5) studies with fewer than 20 patients in their series; (6) case reports; (7) editorial articles; and (8) articles not published in English.

### 2.3 Statistical Analysis

The primary outcome measures of this study were long‐term oncological outcomes, specifically overall survival, disease‐free survival, and recurrence. These outcomes were treated as dichotomous variables and reported as odds ratios (ORs) with 95% confidence intervals (CIs). ORs were calculated based on raw event data from the included RCTs to allow direct comparison between surgical interventions.

Secondary outcomes included operative time, intraoperative blood loss, proximal incision length, number of lymph nodes resected, time to first flatus, and time to first ambulation. These outcomes were expressed as continuous variables and reported as mean differences (MDs) with corresponding 95% CIs.

## 3 Results

### 3.1 Literature Search

In total, 584 articles were identified before 53 duplicate articles were excluded. Thereafter, 531 study titles were screened, before 97 abstracts were reviewed for relevance. All 97 of these studies were eligible for full‐text review and 16 studies met the eligibility criteria and were included in this systematic review and meta‐analysis (Figure 1).

**FIGURE 1:**
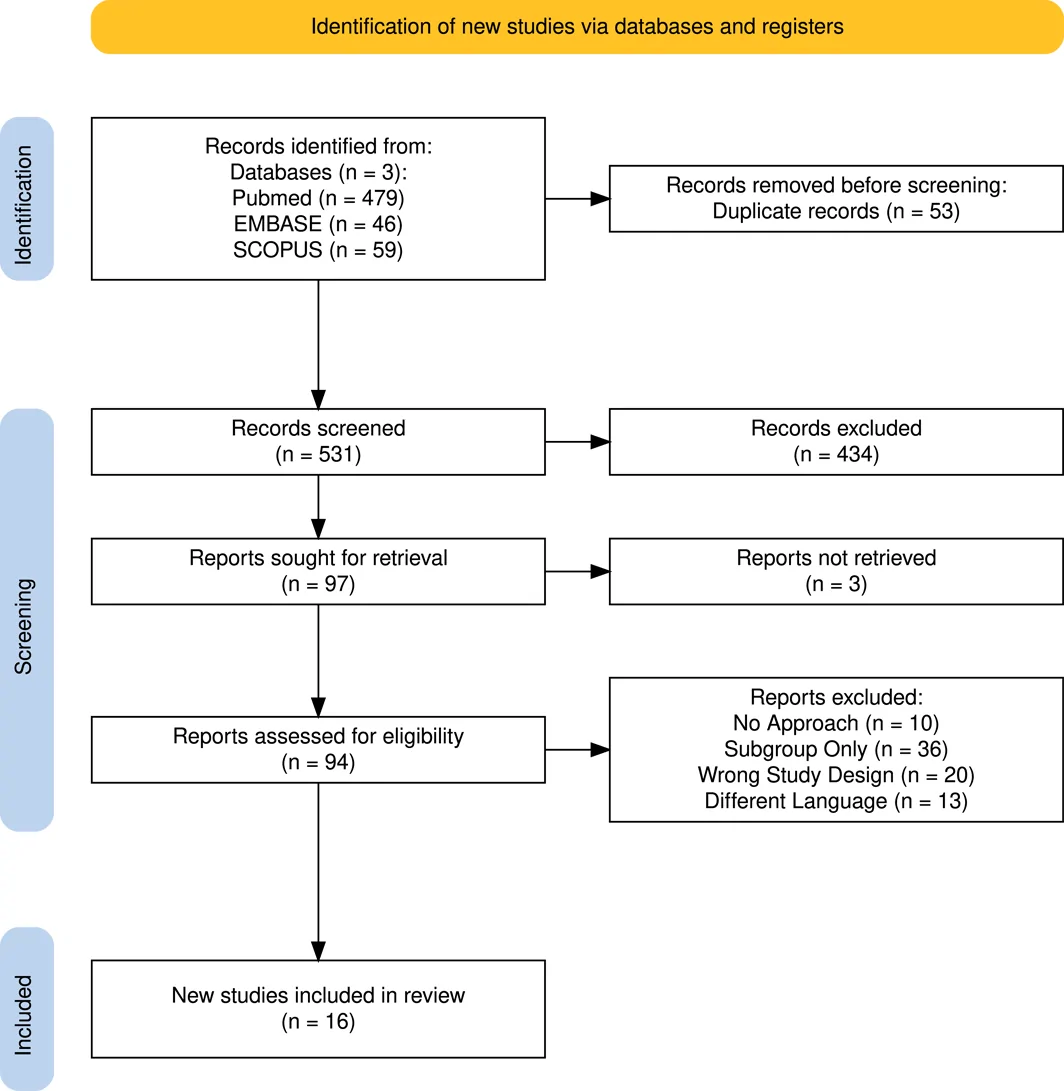
PRISMA flow diagram.

### 3.2 Study and Patient Characteristics

All 16 studies reported outcomes in relation to patients indicated to undergo resection of Siewert II/III AEG (100.0%, 16/16). Publication dates of included studies ranged from 2013 to 2023. Overall, data from 3393 patients were included, of whom 1905 underwent laparoscopic resection (56.15%), 1389 underwent open resection (40.94%) and 99 underwent robotic resection (2.92%). Types of resection between studies varied from Sweet esophagectomy to extended total gastrectomy, and study data for the 16 included studies are depicted in Table 1 whereas peri‐operative information is outlined in Supporting Information S1: Material S1 [13, 14, 15, 16, 17, 18, 19, 20, 21, 22, 23, 24, 25].

**TABLE 1:** Study characteristics.

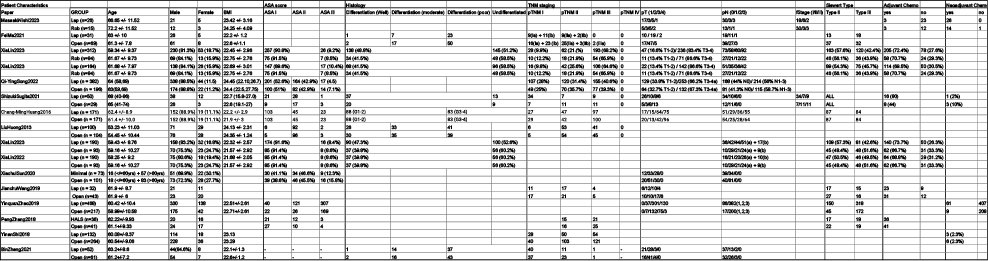

### 3.3 Primary Outcome Measures

#### 3.3.1 Overall Survival

We defined overall survival in this study as the survival from the date of surgery or until death. Overall, 50% of the studies (8/16) reported outcomes related to overall survival (OS). The duration of OS follow‐up varied among studies, with 18.8% of studies reporting outcomes over 3‐years (3/16 studies) and 31.3% of studies over 5‐years (5/16 studies).

Among the studies reporting OS over 3‐years, 78.9% of patients were alive at follow‐up (758/961). OS rates across the three surgical approaches were: Open, 70.8% (260/367); Laparoscopic, 83.6% (484/579); and Robotic, 93.3% (14/15). Compared to the laparoscopic approach, the robotic approach showed similar nonstatistically significant OS (OR 1.17, 95% CI [0.10–14.06]), whereas the open approach showed nonstatistically significant lower OS (OR 0.56, 95% CI [0.41–0.78]) (Figure 2) (Supporting Information S1: Material S2 and S3).

**FIGURE 2:**
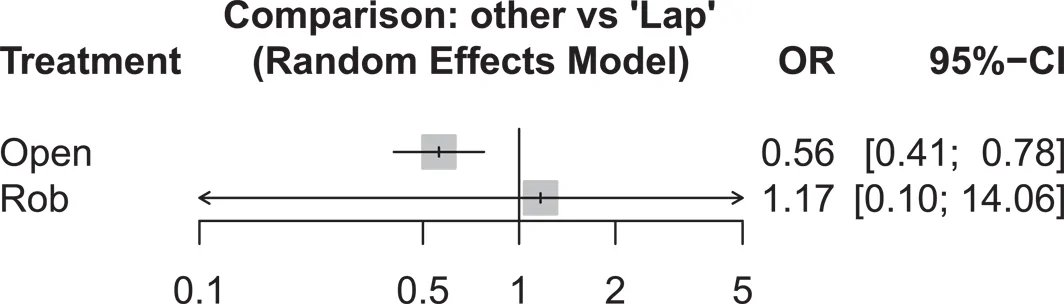
Open and robotic approaches compared to laparoscopic approach (3 year overall survival).

In the studies reporting OS over 5‐years, 41.4% of patients were alive at follow‐up (630/1520). OS rates were: Open, 35.5% (135/380); Laparoscopic, 43.4% (458/1056); and Robotic, 44.0% (37/84). Compared to the laparoscopic approach, the open approach demonstrated a lower but nonstatistically significant OS (OR 0.66, 95% CI [0.39–1.12]), whereas the robotic approach showed a similar OS (OR 1.02, 95% CI [0.41–2.51]) (Figure 3) (Supporting Information S1: Material S4–S6).

**FIGURE 3:**
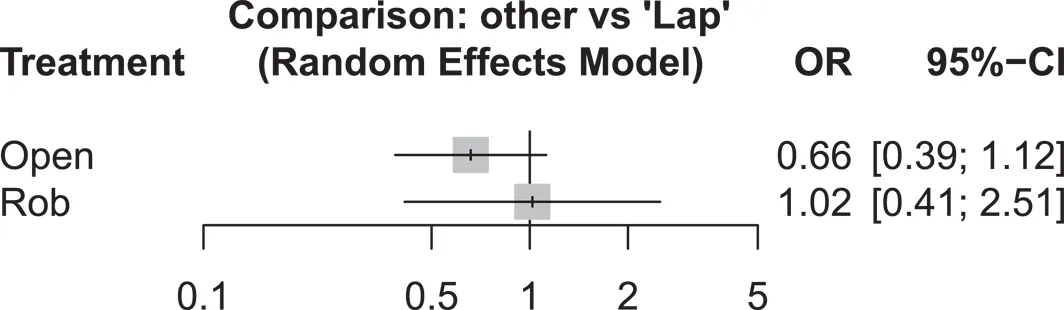
Open and robotic compared to laparoscopic (5 year Overall Survival).

#### 3.3.2 Disease‐Free Survival

We defined disease‐free survival in this study as the disease‐free status of a patient from the day of surgery until recurrence or death. Overall, 31.3% of the studies (5/16) reported outcomes related to disease‐free survival (DFS). The duration of DFS follow‐up varied, with 18.8% (3/16) of studies reporting outcomes over 3‐years and 12.5% (2/16) reporting outcomes over 5‐years.

Among the studies reporting DFS over 3‐years, 72.8% of patients were disease‐free at follow‐up (700/961). DFS rates were: Open, 66.2% (243/367); Laparoscopic, 76.5% (443/573); and Robotic, 93.3% (14/15). Compared to the laparoscopic approach, the open approach showed significant lower DFS (OR 0.69, 95% CI [0.51–0.93]), whereas the robotic approach showed a slightly higher DFS (OR 1.17, 95% CI [0.10–14.06]) (Figure 4) (Supporting Information S1: Material S7 and S8).

**FIGURE 4:**
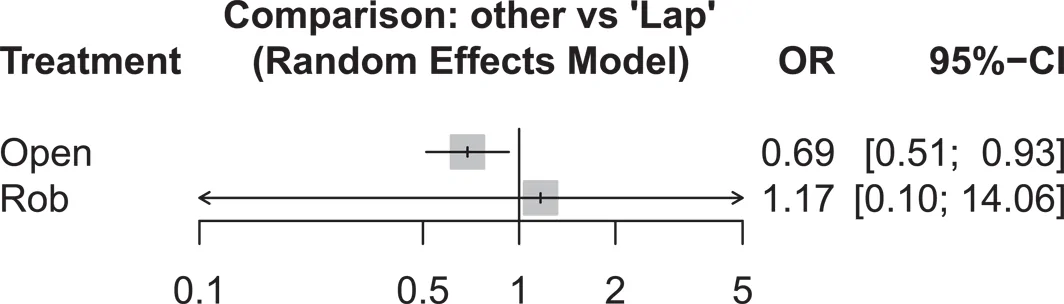
Open and robotic compared to laparoscopic (3‐year disease free survival).

Among the studies reporting DFS over 5‐years, 39% of patients were disease‐free at follow‐up (265/679). DFS rates were: Open, 31.2% (29/93); Laparoscopic, 39.8% (200/502); and Robotic, 42.9% (36/84). Compared to the laparoscopic approach, the open approach demonstrated significantly lower DFS (OR 0.56, 95% CI [0.41–0.78]), whereas the robotic approach again showed a slightly higher DFS (OR 1.17, 95% CI [0.10–14.06]) (Figure 5) (Supporting Information S1: Material S9 and S10).

**FIGURE 5:**
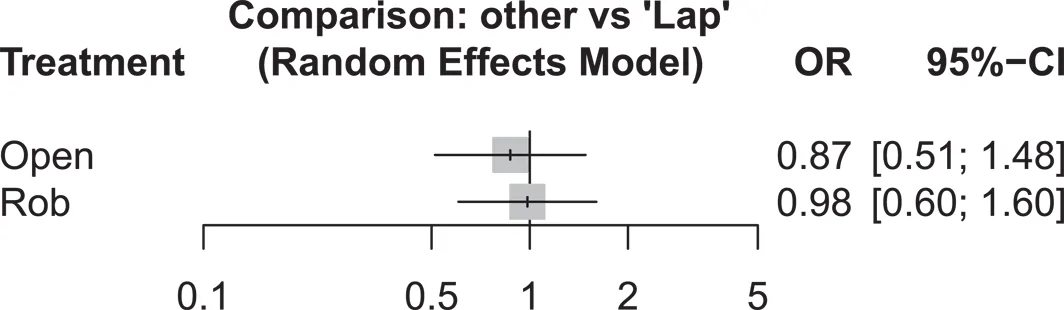
Open and robotic compared to laparoscopic (5‐year disease free survival).

#### 3.3.3 Recurrence

We defined recurrence in this study to be radiological or histological signs of recurrent disease. Overall, 25% of the studies (4/16) reported on recurrence rates. Among all patients included in studies evaluating recurrence, 21.9% experienced disease recurrence (183/835). Recurrence rates were: Open, 36.8% (60/163); Laparoscopic, 17.2% (101/588); and Robotic, 26.2% (22/84).

Compared to the laparoscopic approach, the open approach was associated with a higher recurrence rate, which was not of significant difference (OR 2.29, 95% CI [0.88–5.98]). The robotic approach also showed a higher recurrence rate, though to a lesser extent and not of significant difference (OR 2.00, 95% CI [0.45–8.86]) (Supporting Information S1: Material S11–S13).

### 3.4 Secondary Outcome Measures

#### 3.4.1 Intraoperative Outcomes

##### 3.4.1.1 Operating Time

Overall, 87.5% of the studies (14/16) reported mean operating time. The open approach demonstrated a significantly shorter time (MD −22.34, 95% CI [−39.92 to −4.76]), whereas the robotic approach was associated with a longer time (MD 19.84, 95% CI [−25.87 to 65.55]), but did not reach statistical significance (Supporting Information S1: S14–S17).

##### 3.4.1.2 Blood Loss

Similarly, 87.5% of the studies (14/16) reported intraoperative blood loss. The open approach demonstrated significantly higher blood loss (MD 98.23, 95% CI [65.76 to 130.70]), whereas the robotic approach showed a reduction (MD −13.55, 95% CI [−86.77 to 59.67]). It is noted the reduction of blood loss in robotic approaches did not reach statistical significance (Supporting Information S1: Material S18–S21).

##### 3.4.1.3 Lymph Node Resection

A total of 87.5% of the studies (14/16) reported mean lymph node resection including abdominal and mediastinal resections. Compared to the laparoscopic approach, minimally invasive surgical excision (MISE) was associated with fewer lymph nodes resected (MD −5.03, 95% CI [−6.99 to −3.07]), as was the robotic approach (MD –3.86, 95% CI [−6.49 to −1.24]). The open approach, in contrast, demonstrated a higher number of lymph nodes resected (MD 1.44, 95% CI [0.59 to 2.28]) (Supporting Information S1: Material S22–S25).

##### 3.4.1.4 Proximal Margin Length

Overall, 37.5% of the studies (6/16) evaluated proximal margin length or the distance from the top edge of the tumor to the cut edge of the resected esophagus. Compared to the laparoscopic approach, the open approach showed a nonsignificant difference (MD −0.14, 95% CI [−0.86 to 0.59]), and the robotic approach showed a nonsignificant difference also (MD −0.10, 95% CI [−1.42 to 1.22]) (Supporting Information S1: Material S26–S28).

### 3.5 Postoperative Outcomes

#### 3.5.1 Time to First Flatus

Overall, 37.5% of the studies (6/16) assessed time to first flatus. Compared to the laparoscopic approach, the open approach was associated with a nonsignificant increased length in time (MD 1.02, 95% CI [0.18 to 1.85]), whereas the robotic approach showed a statistically nonsignificant shorter time (MD −0.40, 95% CI [−2.27 to 1.47]) (Supporting Information S1: Material S29–S31).

#### 3.5.2 Time to First Ambulation

Overall, 25% of the studies (4/16) evaluated time to first ambulation. Compared to the laparoscopic approach, the open approach was associated with a nonsignificant longer time (MD 1.21, 95% CI [0.44 to 1.97]), whereas the robotic approach showed a statistically nonsignificant shorter time (MD −0.30, 95% CI [−1.60 to 1.00]) (Supporting Information S1: Material S32–S34).

### 3.6 Risk of Bias

Overall, the included retrospective cohort and case–control studies generally scored well on the Newcastle–Ottawa Scale, with most achieving 8–9/9 points, indicating strong selection of participants, appropriate comparability, and adequate outcome assessment. However, the predominance of retrospective, single‐center designs inherently increases the risk of selection bias and limits generalizability. Although many studies employed methods such as propensity score matching to minimize confounding, residual and unmeasured confounding cannot be excluded. In some cases, incomplete or unspecified follow‐up rates raise the potential for attrition bias, and variations in study periods introduce the possibility of heterogeneity due to evolving surgical techniques and perioperative care.

## 4 Discussion

This network meta‐analysis evaluated the long‐term oncologic outcomes and surgical efficacy of open, laparoscopic, and robotic approaches in the management of Siewert type II/III adenocarcinoma of the AEG. Drawing from 16 independent observational studies, this analysis demonstrates the noninferiority of robotic surgery compared to laparoscopy in terms of long‐term survival, indicating that both minimally invasive approaches provide similar oncological safety for such patients. In addition, the data previously described the advantages of minimally invasive surgery, in particular robotic approaches, where reduced intraoperative blood loss and shorter hospital stays have been described [26, 27, 28, 29, 30]. Thus, these findings support the expanding role of laparoscopic‐ and robotic‐assisted surgery as effective alternatives to open surgery for curative treatment of Siewert II/III AEG.

The current analysis of OS reveals a consistent trend toward significant in favor of minimally invasive techniques. Overall, 3‐year OS was 83.6% for laparoscopic‐assisted and 93.3% for robotic‐assisted surgery, compared with 70.8% for open surgery; however, it is important to acknowledge that no formal matching occurred in the data for age and stage. Thus, these findings suggest a potential noninferiority of robotic approaches compared to conventional methods in the curative management of Siewert II/III AEG, a trend aligning with existing literature. A previous meta‐analysis reported a significant improvement in 5‐year OS with laparoscopy over open surgery (hazard ratio (HR): 1.30; 95% CI: 1.06–1.60; *p* = 0.01), whereas an original retrospective cohort study showed a significant survival advantage for laparoscopic resection (HR = 0.68; 95% CI: 0.58–0.81; *p* < 0.00001) [9, 10, 14, 16, 19]. This could be attributed to a few factors that are difficult to measure objectively including surgical expertise, institutional training, or advancing technologies. Minimally invasive operating can give a surgeon a better field of vision within their operating field, especially when taking into account robotic surgery which could also attribute to favorable outcomes in these cohorts.

In this study, outcomes in relation to DFS demonstrated a similar pattern. Three‐year DFS rates were 76.5% for laparoscopic‐assisted compared to 93.3% for robotic‐assisted approaches. This data indicates a durable oncologic benefit with both minimally invasive strategies, reinforcing their efficacy in establishing disease control for the majority. Similar to with OS, comparable findings were reported in recent literature: A 2024 meta‐analysis demonstrated a significant 5‐year DFS advantage with laparoscopy over open surgery (HR = 0.68; 95% CI: 0.59–0.77; *p* < 0.00001) [10, 14, 16, 19], whereas a 2022 study similarly showed improved DFS with laparoscopy (RR = 1.31; 95% CI: 1.07–1.60; *p* = 0.01) [9].

Conversely to OS and DFS, recurrence data showed a modest, nonsignificant increase in the open (36.8%) and robotic (26.2%) groups compared to laparoscopy (17.2%). Although this may appear to challenge the overall trend, it likely reflects the larger cancers necessitating open resection and their propensity to develop recurrence as a consequence, though heterogeneous follow‐up intervals across centers and the differences in cohort size further complicates interpretation. Moreover, the absence of high‐quality data availability from prospective randomized trials outlining the true recurrence data comparing open, laparoscopic, and robotic surgery limits definitive conclusions which can be drawn in this context.

Although long‐term oncologic results for laparoscopy and robotics are comparable, perioperative parameters seem to differentiate these two approaches: In this analysis, robotic‐assisted surgery was associated with a significantly lower intraoperative blood loss, faster return of bowel function, earlier time to ambulation, and a shorter hospital stay when compared to laparoscopic‐assisted approaches. These outcomes have been well described in the context of other cancers and are not surprising findings in this context [26, 27, 28, 29, 30].

Although the robotic approach offers certain perioperative advantages, it is important to consider its limitations. Robotic surgery necessitates specialized training and is associated with a significant learning curve, potentially affecting both operative efficiency and widespread accessibility. In this analysis, operative time was significantly longer for robotic procedures compared to both laparoscopic and open techniques. When considered alongside the broadly equivalent long‐term oncologic outcomes and only modest gains in short‐term recovery, these findings raise valid concerns regarding the routine adoption of robotic surgery, particularly in resource‐limited settings or where surgical expertise is still developing. Time to first flatus and time to first ambulation were also shorter in the robotic cohort, which are quite dependent on institutional protocol, surgeons’ preference, and the availability of nursing staff and physiotherapists which may skew results and are not robust in a measure of surgical outcomes. Notably, factors such as setup time, docking, and intraoperative machine management were not assessed in this analysis and warrant investigation to fully understand their impact on operative workflow and overall efficiency.

We have included Siewert II and III adenocarcinomas together, as these subtypes represent the majority of real‐world GOJ cancers where surgical decision‐making remains most controversial and where transabdominal approaches (total gastrectomy ± lower esophagectomy) are commonly employed, unlike the transthoracic approach typically required for Siewert I. These subtypes are frequently grouped in clinical practice for perioperative management and lymphadenectomy planning due to their overlapping lymphatic drainage patterns to both gastric and mediastinal nodes [5, 6, 8]. However, this approach must be interpreted with caution given their biological heterogeneity: Siewert II tumors extend above the GOJ (1–2 cm distal), behave more like esophageal cancers with higher rates of nodal metatasis and mediastinal spread, whereas Siewert III tumors (2–5 cm distal) are classified as gastric cancers with distinct prognostic and treatment profiles [5, 6, 8]. Consequently, their differing optimal surgical strategies—transthoracic esophagectomy for adequate II margins versus transhiatal total gastrectomy for III—may mask technique‐specific outcomes in our pooled analysis [6, 8].

This analysis is subject to several limitations. The majority of studies included were conducted in Eastern countries, primarily China and Japan, which may limit translation to Western healthcare settings due to differences in genetic predispositions, institutional expertise and practice, consensus guidelines, and the accepted prescription of neoadjuvant and adjuvant therapeutics. Furthermore, the studies demonstrated considerable variation in follow‐up durations, potentially impacting the accuracy and comparability of long‐term outcome data. Many of the included studies were also single‐center in design, introducing certain biases related to surgeon‐specific experience, institutional practices, and the presence of specialized centers, thereby limiting external validity. It is also noted that of the research included in this systematic review, when confirming negative margins in these patients, only 2 papers confirmed using intraoperative frozen section, which makes it difficult to confirm completely R0 resection rates in the included papers and may introduce bias and skew results. As previously mentioned, the predominance of retrospective, single‐center designs inherently increases the risk of selection bias and limits generalizability.

In conclusion, this analysis demonstrates equipoise of robotic approaches to the resection of Siewert type II/III adenocarcinoma of the esophagogastric junction in terms of long‐term oncological outcomes, while also highlighting promising results in the perioperative period. Notably, factors related to setup time and machine management in robotic surgery were beyond the scope of this review and could not be assessed. Furthermore, our findings support the superiority of laparoscopic techniques over open surgery with respect to long‐term oncological outcomes. Based on these results, we recommend further prospective studies to more comprehensively evaluate both the short‐ and long‐term effectiveness of robotic surgery as a treatment modality for AEG.

## Author Contributions

**Connor J. Colson:** investigation, writing – original draft, writing – review and editing, methodology, project administration, data curation, resources, validation, formal analysis, conceptualization. **Matthew G. Davey:** conceptualization, methodology, software, formal analysis, supervision, writing – review and editing, resources, data curation. **Noel E. Donlon:** supervision, writing – review and editing, conceptualization, resources, data curation.

## Funding

The authors have nothing to report.

## Ethics Statement

The authors have nothing to report.

## Consent

All participants provided informed consent to partake in the studies included in this review.

## Conflicts of Interest

The authors declare no conflicts of interest.

## Permission to Reproduce Material From Other Sources

The authors have nothing to report.

## Supporting information


Supporting Information S1

## Data Availability

Data can be made available upon reasonable request from the corresponding author.
