# Supplementary material for: Comparing Minimally‐Invasive and Open Approaches to Siewert II/III Gastro‐Esophageal Junction Adenocarcinoma—A Systematic Review and Network Meta‐Analysis
Source: World J Surg. 2026 Sep 6;50(10):3024–31. doi: 10.1002/wjs.70528 (PMC13662513; doi:10.1002/wjs.70528)
Supplement: Supplementary file 1 [file WJS-50-3024-s001.docx]

Supplementary Material 1. Patient Characteristics

Supplementary Material 2. Network Plot of 3 year Overall Survival

Supplementary Material 3. Individual Study results 3 year Overall Survival

Supplementary Material 4. Network Plot 5 year Overall Survival

Supplementary Material 5. Individual Study results 5 year Overall Survival

Supplementary Material 6. Summary Forest plot 5 year Overall Survival

Supplementary Material 7. Network Plot 3 year Disease Free Survival

Supplementary Material 8. Individual Study results 3 year Disease Free Survival

Supplementary Material 9. Network Plot 5 year disease free survival

Supplementary Material 10. Individual Study results 5 year disease free survival

Supplementary Material 11. Open and Robotic Approaches compared to Laparoscopic Approach (Recurrence)

Supplementary Material 12. Network Plot Recurrence

Supplementary Material 13. Individual study results Recurrence

Supplementary Material 14. Open, Robotic and Minimally invasive surgical excision compared to Laparoscopic (Operative Time)

Supplementary Material 15. Individual Study Results (Operative Time)

Supplementary Material 16. Summary Forest Plot (Operative Time)

Supplementary Material 17. Network Plot (Operative Time)

Supplementary Material 18. Open, Robotic and Minimally invasive surgical excision compared to Laparoscopic (Intra-operative blood loss)

Supplementary Material 19. Individual Study Results (Intra-operative blood loss)

Supplementary Material 20. Summary Forest Plot (Intra-operative blood loss)

Supplementary Material 21. Network Plot (Intra-operative blood loss)

Supplementary Material 22. Open, Robotic and Minimally invasive surgical excision compared to Laparoscopic (Lymph node resection)

Supplementary Material 23. Individual Study Results (Lymph node resection)

Supplementary Material 24. Summary Forest Plot (Lymph node resection)

Supplementary Material 25. Network Plot (Lymph node resection)

Supplementary Material 26. Open and Robotic compared to Laparoscopic (Proximal margin)

Supplementary Material 27. Individual Study results (Proximal margin)

Supplementary Material 28. Network Plot (Proximal margin)

Supplementary Material 29. Open and Robotic compared to Laparoscopic (Time to First Flatus)

Supplementary Material 30. Individual Study Results (Time to First Flatus)

Supplementary Material 31. Network plot (Time to First Flatus)

Supplementary Material 32. Open and Robotic compared to Laparoscopic (Time to First Ambulation)

Supplementary Material 33. Individual Study Results (Time to First Ambulation)

Supplementary Material 34. Network Plot (Time to First Ambulation)
